# Supplementary figures and images for: Comprehensive analysis of miRNA and protein profiles within exosomes derived from canine lymphoid tumour cell lines
Source: PLoS One. 2019 Apr 29;14(4):e0208567. doi: 10.1371/journal.pone.0208567 (PMC6488050; doi:10.1371/journal.pone.0208567)

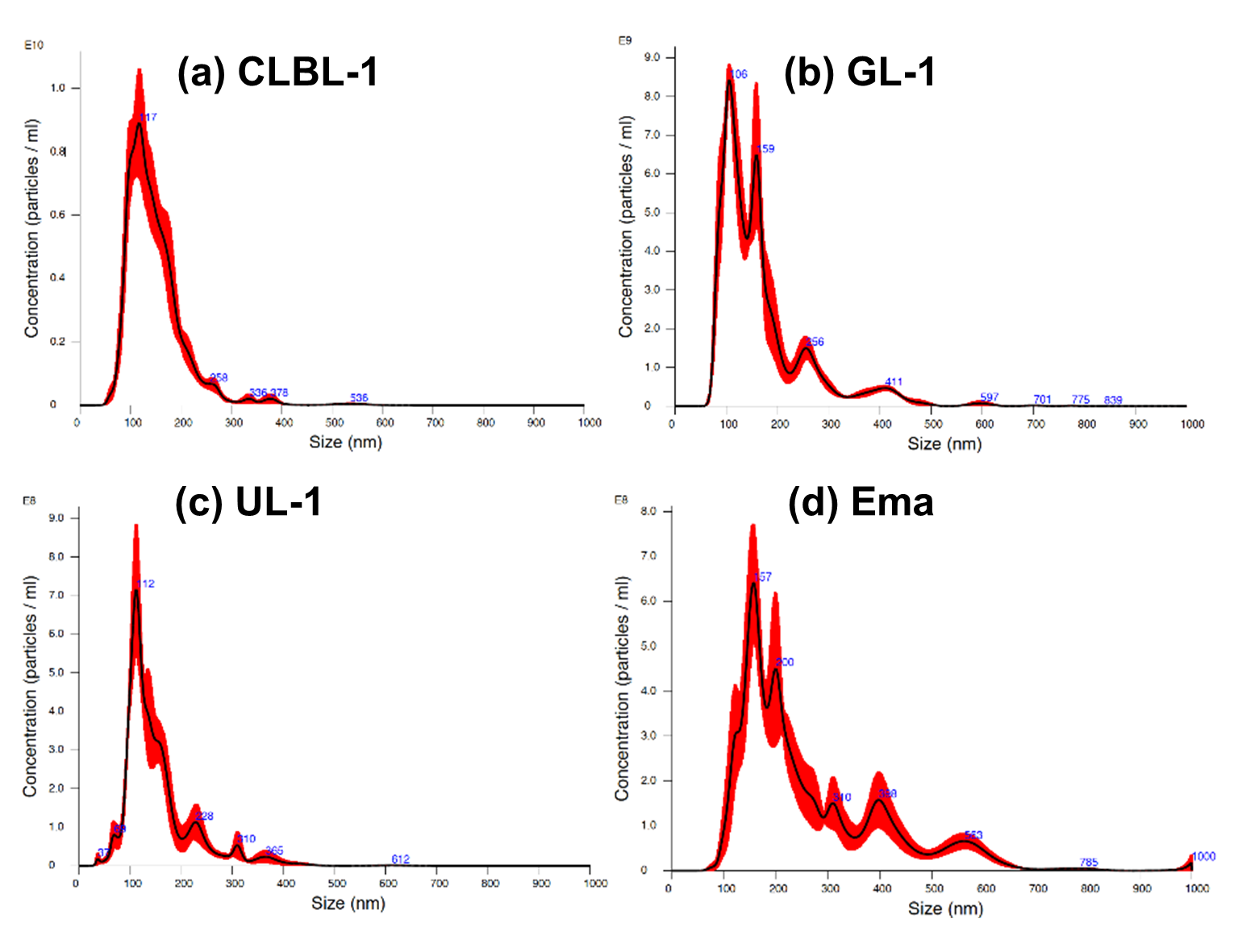

Supplement: S1 Fig — Size distributions of exosomes of CLBL-1 (a), GL-1 (b), UL-1 (c), and Ema (d). (TIF) [file pone.0208567.s005.tif]

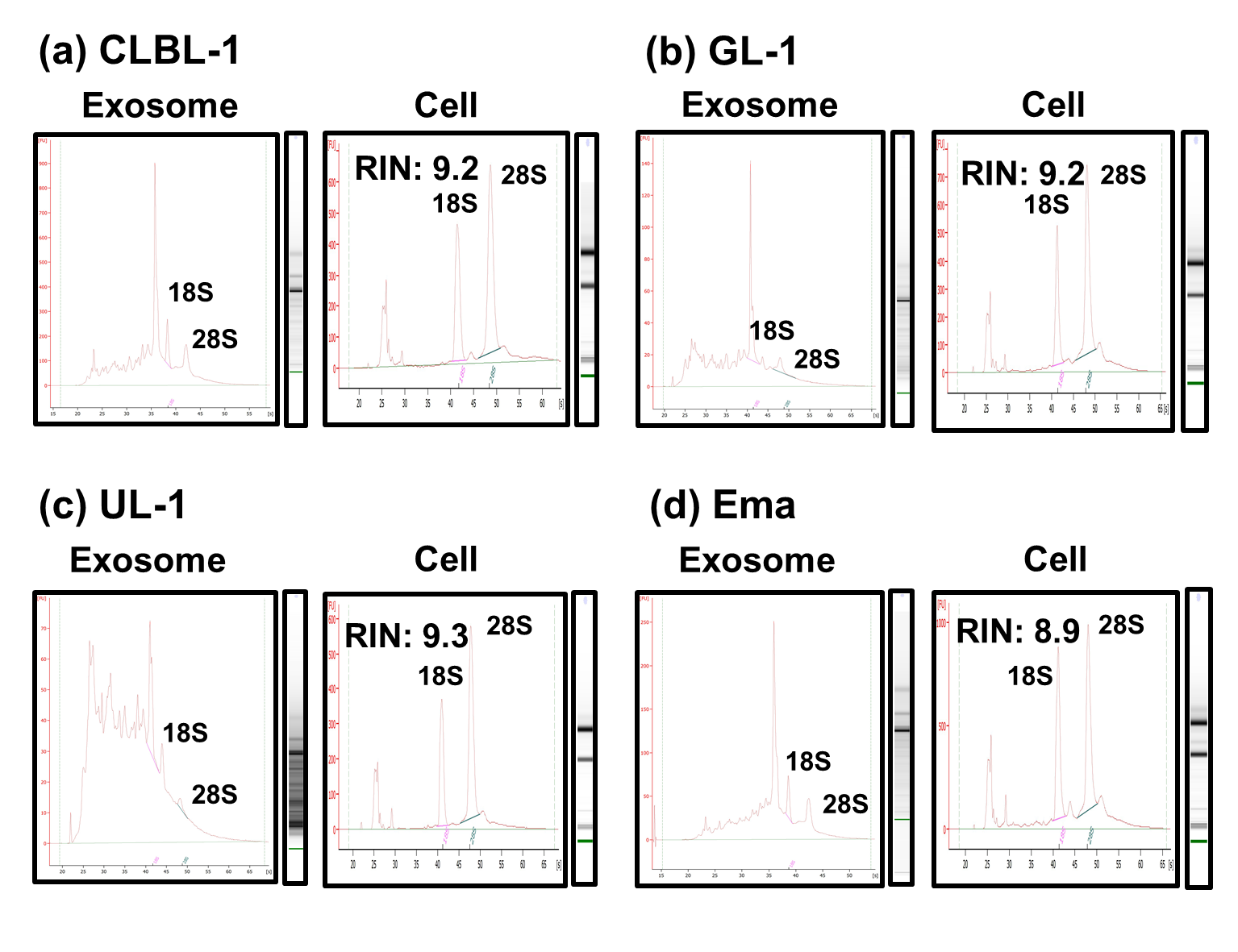

Supplement: S2 Fig — “18S” indicates the peak corresponds to 18S ribosomal RNA, and “28S” indicates that corresponds to 28S ribosomal RNA. (TIF) [file pone.0208567.s006.tif]

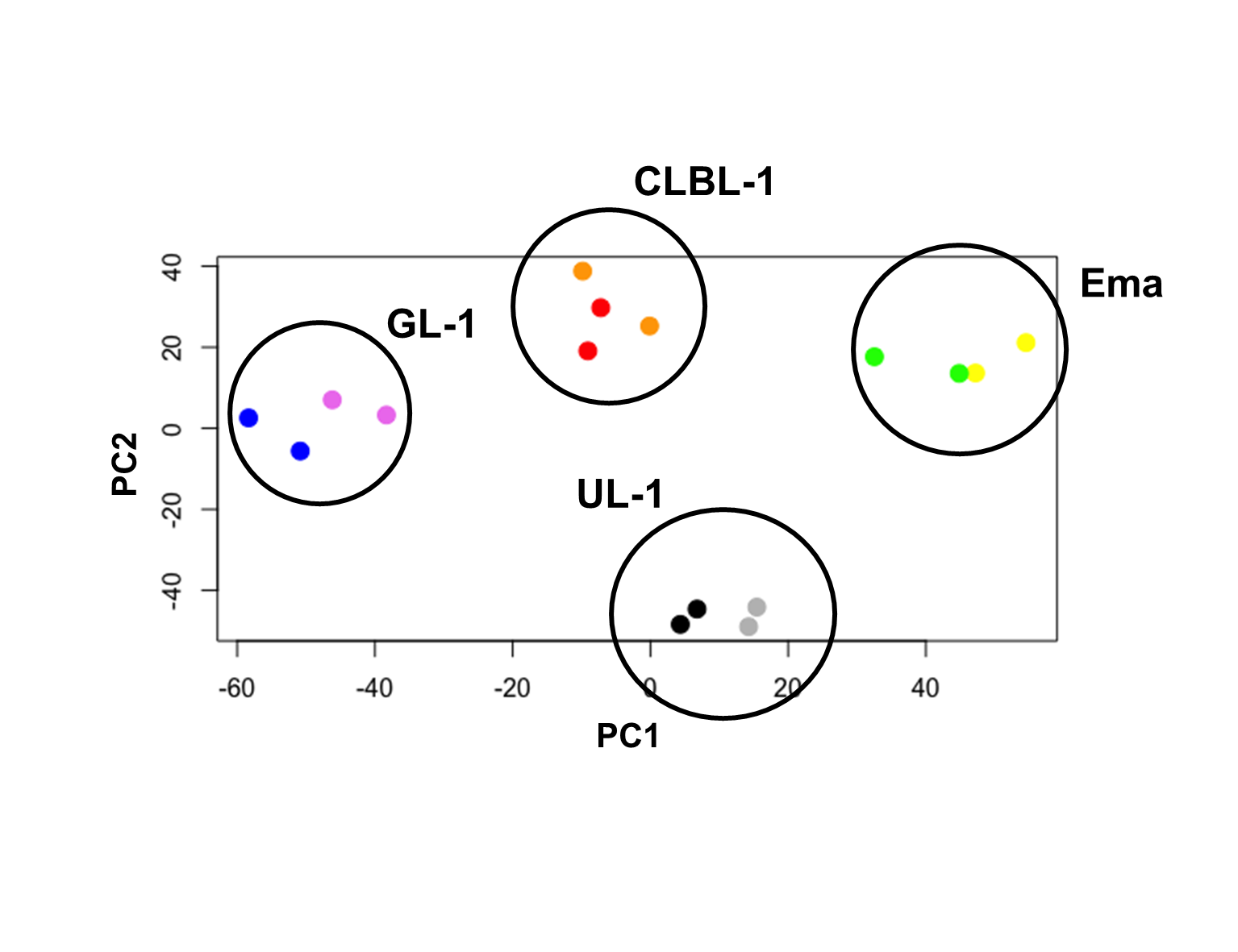

Supplement: S3 Fig — Exosomes and parent cells clustered similarly for each cell line and the profiles are different among cell lines. Orange dots (exosomes) and red dots (parent cells) correspond to CLBL-1, violet dots (exosomes) and blue dots (parent cells) to GL-1, grey dots (exosomes) and black dots (parent cells) to UL-1, and yellow dots (exosomes) and green dots (parent cells) to Ema. (TIF) [file pone.0208567.s007.tif]

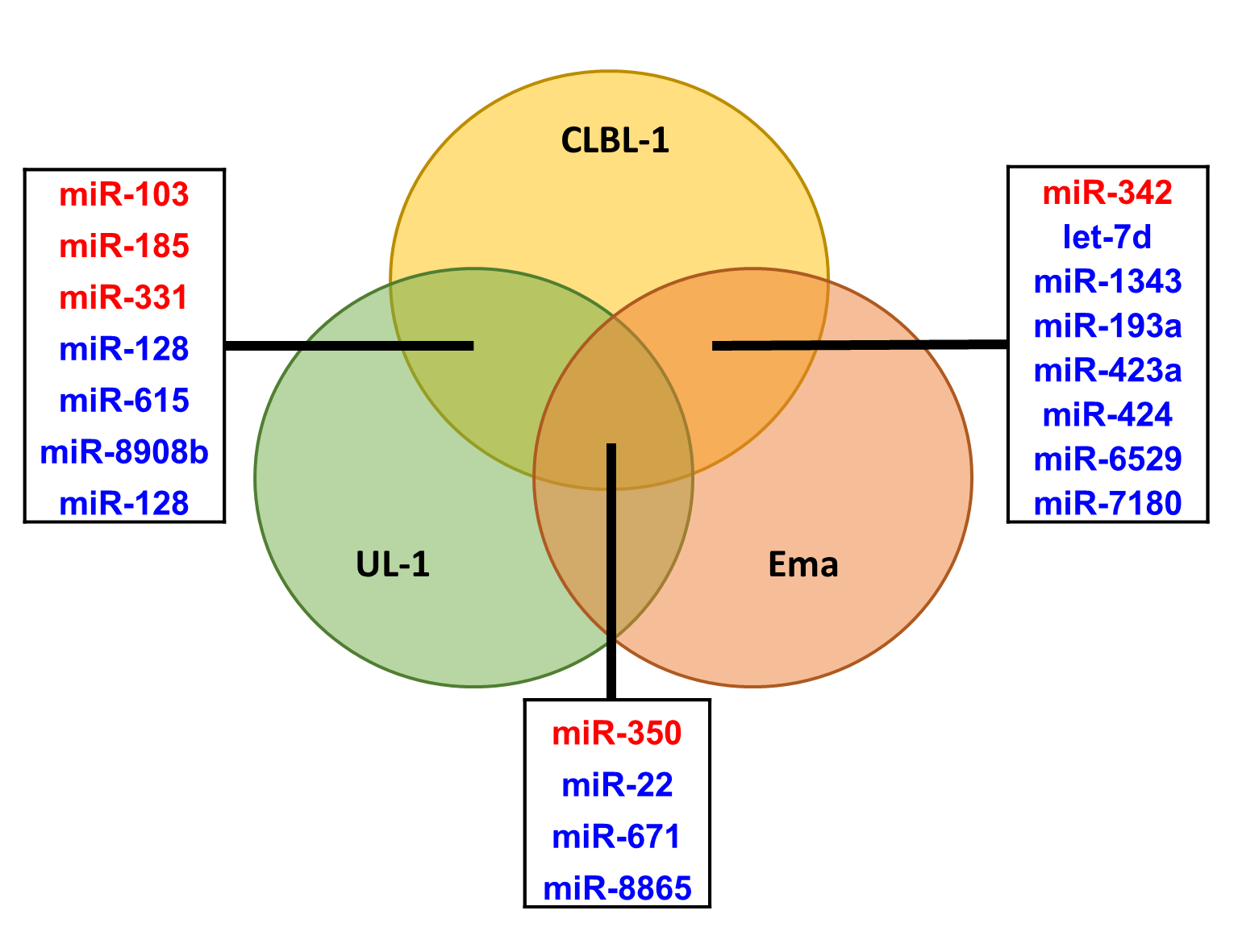

Supplement: S4 Fig — The names of miRNAs whose amounts were significantly larger in exosomes than parent cells are shown in red, and those whose amounts were significantly smaller in exosomes than parent cells are shown in blue. (TIF) [file pone.0208567.s008.tif]

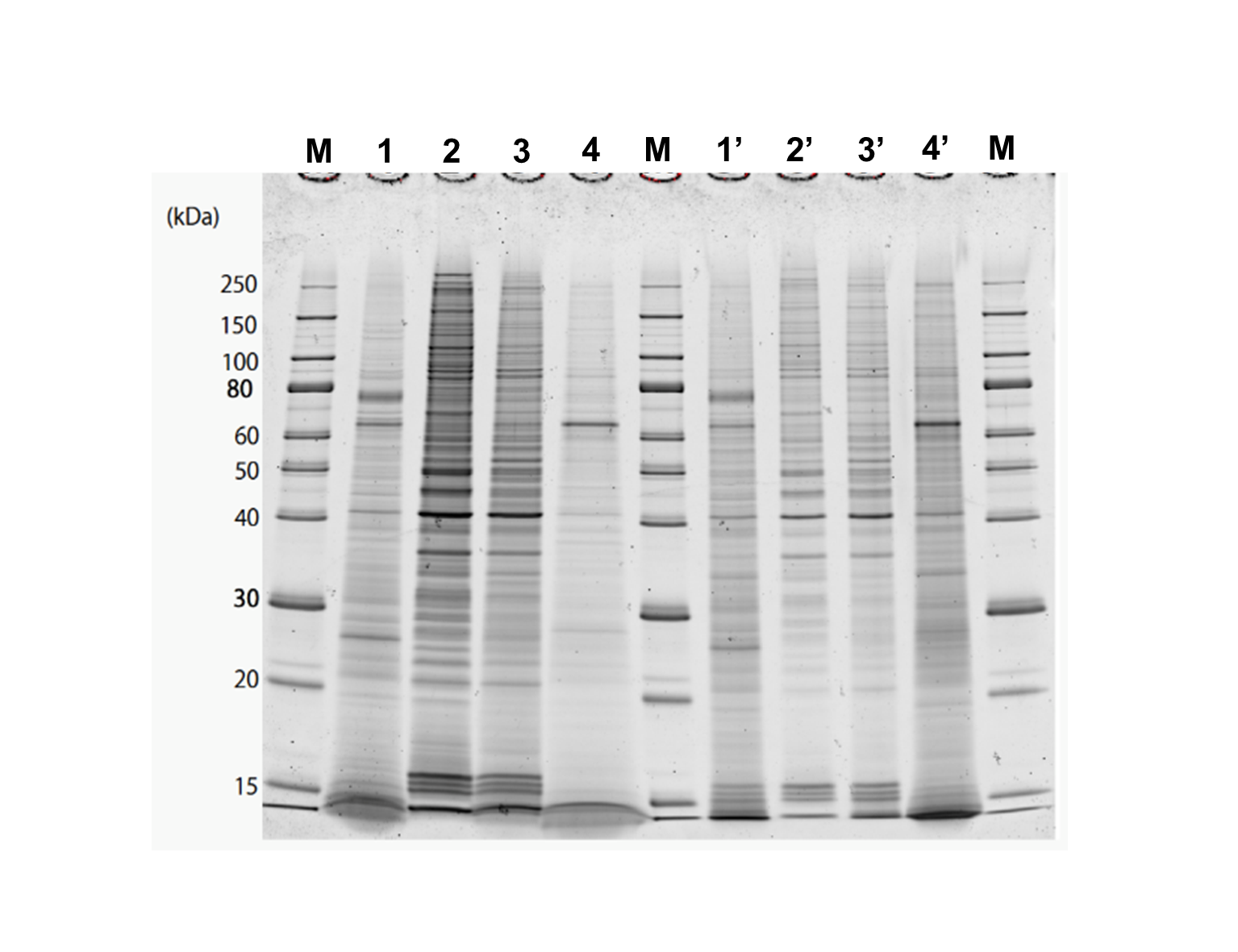

Supplement: S5 Fig — Lane M is the protein ladder. Lanes 1–4 correspond to the exosomal proteins extracted from CLBL-1, GL-1, UL-1, and Ema, respectively, and lanes 1’-4’ correspond to exosomal proteins precipitated with trichloroacetic acid extracted from CLBL-1, GL-1, UL-1, and Ema, respectively. (TIF) [file pone.0208567.s009.tif]

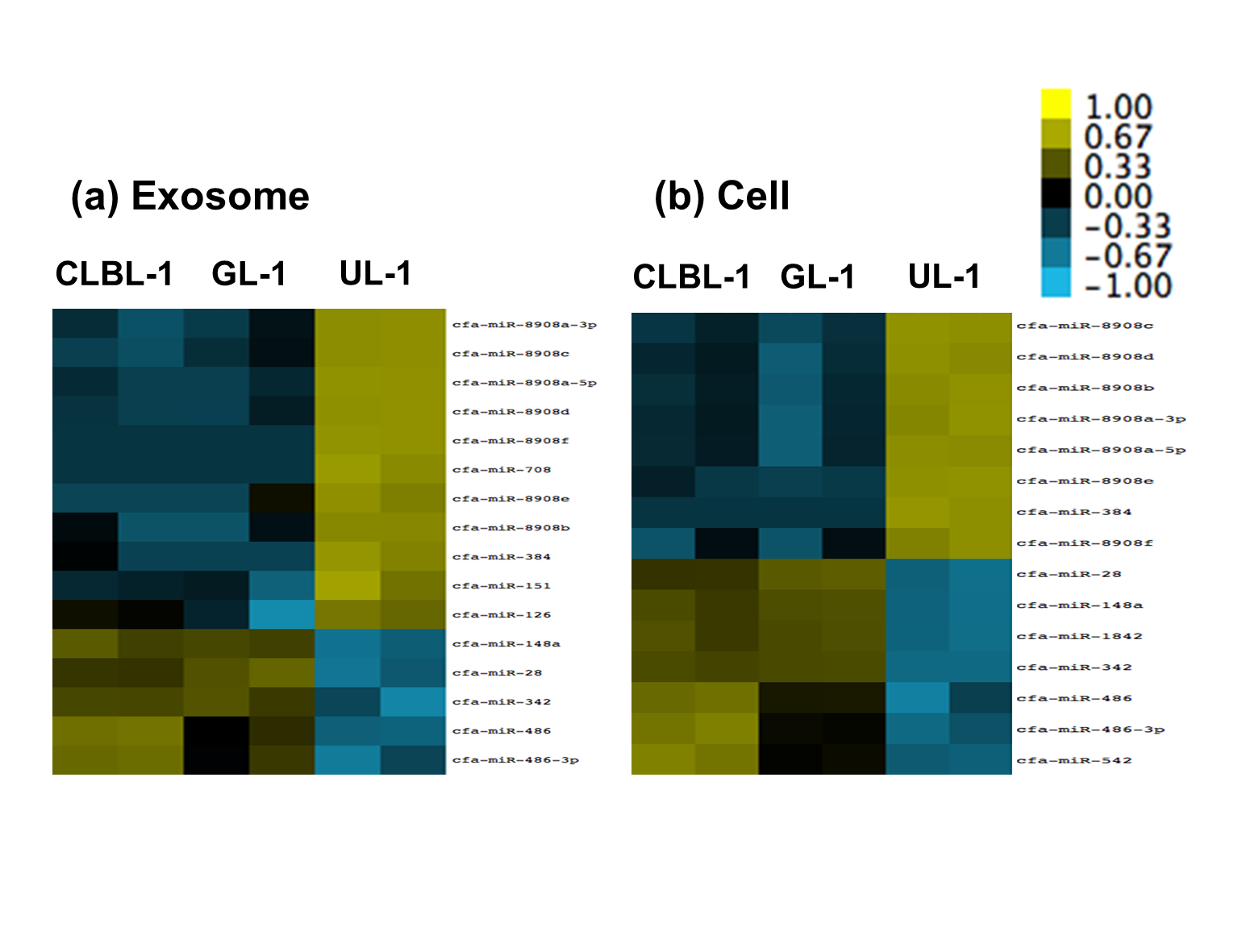

Supplement: S6 Fig — In exosomes (a), the amounts of 11 miRNAs were significantly lower in VCR-S cell lines than VCR-R cell line, and those of 5 miRNAs were significantly higher in VCR-S cell lines than VCR-R cell line. In parent cells (b), the amounts of 8 miRNAs were significantly lower in VCR-S cell lines than VCR-R cell line, and those of 7 miRNAs were higher in VCR-S cell lines than VCR-R cell line. (TIF) [file pone.0208567.s010.tif]

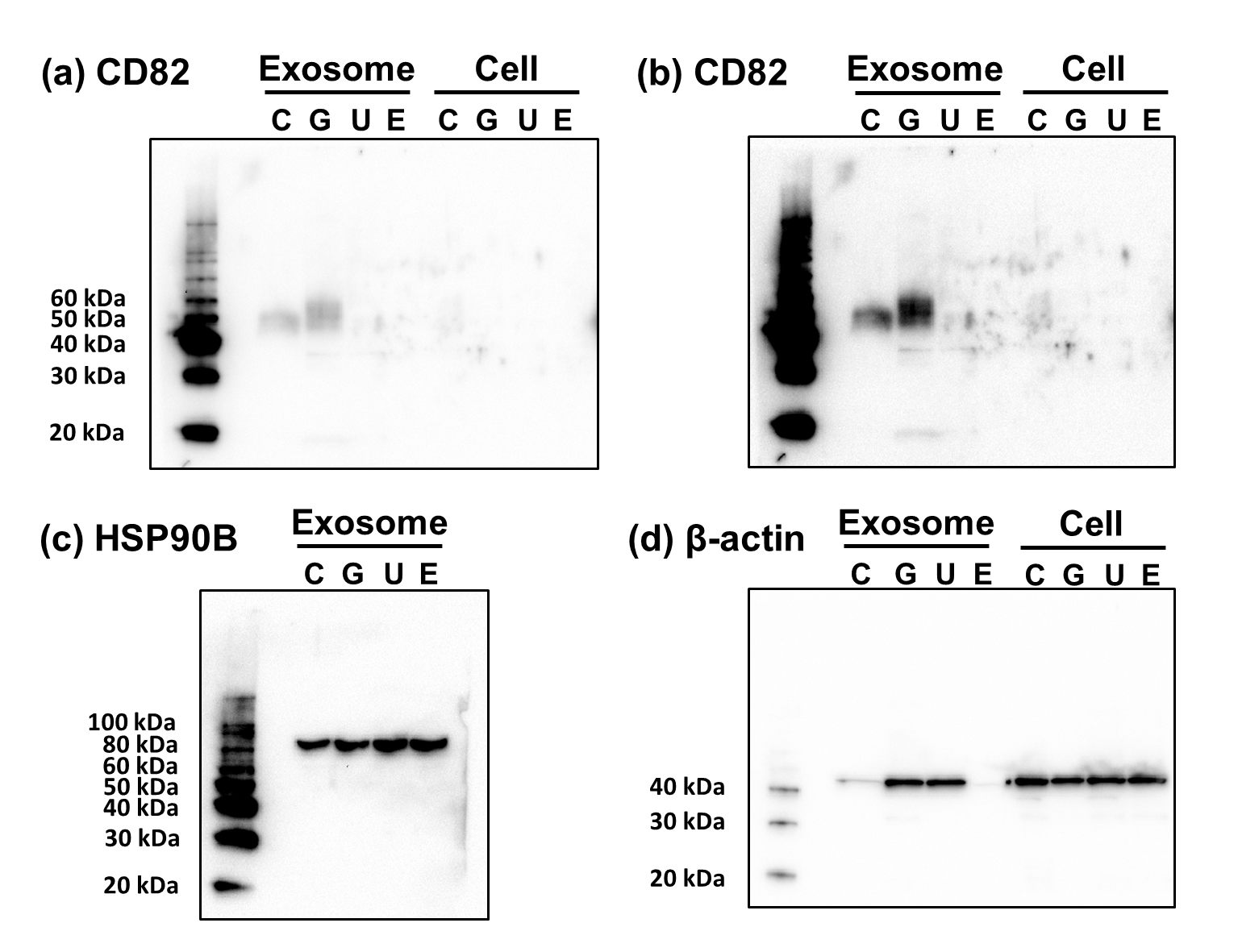

Supplement: S7 Fig — The figures of the same membrane were shown in (a) and (b), but exposure times were different between these figures. In Fig 5, the figures that show the detection of CD82 within exosomes and parent cells were cropped from the different parts of (b). The figures of detection of HSP90B and β-actin were cropped from (c) and (d), respectively. (TIF) [file pone.0208567.s011.tif]
